# 2-Aminoethoxydiphenyl Borate Potentiates CRAC Current by Directly Dilating the Pore of Open Orai1

**DOI:** 10.1038/srep29304

**Published:** 2016-07-04

**Authors:** Xiaolan Xu, Sher Ali, Yufeng Li, Haijie Yu, Mingshu Zhang, Jingze Lu, Tao Xu

**Affiliations:** 1 National Key Laboratory of Biomacromolecules, Institute of Biophysics, Chinese Academy of Sciences, Beijing 100101, China; 2 University of Chinese Academy of Science, Beijing 100049, China; 3 College of Life Science, Sichuan Normal University, Chengdu 610101, China; 4 Department of Physiology and Biophysics, University of Washington, Seattle, WA, USA; 5 Key Laboratory of RNA Biology, Institute of Biophysics, Chinese Academy of Sciences, Beijing 100101, China

## Abstract

2-Aminoethoxydiphenyl borate (2-APB) elicits potentiation current (*I*_*p*_) on Ca^2+^ release-activated Ca^2+^ (CRAC) channels. An accurate investigation into this modulation mechanism would reveal how STIM1-dependent channel gating is enhanced, and benefit the future immune enhancer development. Here, we directly probed the pore diameter of CRAC channels and found that 2-APB enlarged the pore size of STIM1-activated Orai1 from 3.8 to 4.6 Å. We demonstrated that ions with small sizes, i.e., Ca^2+^ and Na^+^, mediated prominent 2-APB-induced *I*_*p*_ on the wildtype (WT) Orai1 channels of narrow pore sizes, while conducted decreased or no *I*_*p*_ on Orai1-V102C/A/G mutant channels with enlarged pore diameters. On the contrary, large Cs^+^ ions blocked the WT channels, while displayed large 2-APB induced *I*_*p*_ on pore-enlarged Orai1-V102C/A/G mutant channels, and the potentiation ratio was highest on Orai1-V102C with an intermediate pore size. Furthermore, we showed that 2-APB potentiated Cs^+^ current on constitutively active Orai1-V102C/A/G mutants independent of STIM1. Our data suggest that 2-APB directly dilates the pore of open Orai1 channels, both ion size and pore diameter jointly determine the amplitude of *I*_*p*_ on CRAC channels, and the generation of *I*_*p*_ requires the open state of Orai1, not STIM1 itself.

The Ca^2+^ release-activated Ca^2+^ (CRAC) channels play an essential and specific role in immune system. Ca^2+^ entry through CRAC channels is the key to triggering lymphocyte activation, proliferation^1^, and mast cell degranulation^2^. A loss-of-function mutation in Orai1, the pore-forming subunit of CRAC channels^3^, causes severe human immune deficiency by abrogating the CRAC channel function^4^, while spares major organs and other physiological systems from impairment^5^. Mutations in STIM1, which is the CRAC channel sensor of endoplasmic reticulum luminal Ca^2+^ concentration^6^,^7^, also cause immunodeficiency syndrome and other disorders^5^,^8^. Given their essential and specific role in human immunity, CRAC channels have emerged as attractive candidates for the development of novel therapeutics to modulate the magnitude of immune response and treat immune disorders^9^.

The pharmacological modulation of CRAC current (*I*_*CRAC*_) has two opposite directions: inhibition and enhancement. By enhancing *I*_*CRAC*_, the immune reaction could be boosted, which might benefit patients with weak immune activity. A promising immune enhancer should work at the right time when the CRAC channels are activated during an immune response, and the properties of enhanced current should be similar to those of native *I*_*CRAC*_ to prevent aberrant ion flow from interfering with the normal physiological reaction. The hallmarks of *I*_*CRAC*_ include several fingerprint properties, such as 1) an extraordinarily high selectivity for Ca^2+^ over Na^+^; 2) a very low permeability to large cations such as Cs^+^; 3) a current-voltage (*I-V*) profile with inward rectification and a positive reversal potential (*V*_*rev*_) of ~50 mV; and 4) an extremely narrow pore diameter of 3.8 Å^10^,^11^. High Ca^2+^ selectivity is not an immutable property of the open CRAC channels but is strictly regulated by the gating of STIM1^12^. It should be noted that CRAC channels exhibit high Ca^2+^ selectivity only in Ca^2+^-containing solutions and readily conduct small monovalent ions such as Na^+^ in divalent-free (DVF) solutions.

Among a range of CRAC channel modulators^13^,^14^, 2-APB is probably the best studied compound, with multiple effects including the activity of gating enhancer. It has been reported that on native immune cells, a high dose of 2-APB initially potentiated *I*_*CRAC*_ (denoted as *I*_*p*_) and then completely inhibited *I*_*CRAC*_. 2-APB-elicited *I*_*p*_ shared similar properties with *I*_*CRAC*_, i.e., *I-V* profile, high Ca^2+^ selectivity, blocking by CRAC channel inhibitors, and only developed after Ca^2+^ store depletion; therefore, it has been suggested that 2-APB-elicited *I*_*p*_ is *I*_*CRAC*_ itself^15^. After the discovery of Orai1 and STIM1 as two essential components of the CRAC channels, the complex effects of 2-APB were investigated and compared among the Orai family (including Orai1, Orai2, and Orai3)^16^,^17^,^18^. A high dose of 2-APB exhibits prominent agonistic activity on Orai3, and to a much less degree on STIM1-free Orai1 channels^16^,^18^. However, this direct-gating effect of 2-APB on Orai3 and Orai1 was characterized by biphasic inwardly and outwardly rectified currents and differed from potentiation effect in many aspects, such as 1) loss of high Ca^2+^ selectivity and altered *I-V* profile; 2) increased permeability to Cs^+^ due to enlarged pore diameter of Orai3^19^,^20^; 3) STIM1-bound channels, especially Orai1, resist this 2-APB direct gating effect^21^, whereas the generation of *I*_*p*_ is STIM1-activation dependent; 4) different dose requirements: the reported EC_50_ for 2-APB-induced *I*_*p*_ was 3.1 μM for native CRAC channels^15^ and was 4 μM for STIM1-activated Orai1 channels^18^, while the reported EC_50_ for 2-APB-direct gating on Orai1 alone was 20 ± 1 μM, and that for Orai3 alone was 14 ± 4 μM^18^.

Under physiological conditions, a direct immune agonist would induce non-specific immune responses, whereas immune enhancers only boost the specific and necessary immune reaction. Because 2-APB-induced *I*_*p*_ requires store depletion as a prerequisite and possesses properties, especially high Ca^2+^ selectivity, similar to those of *I*_*CRAC*_ on native immune cells, which dominantly express Orai1 and STIM1, it thus serves as an ideal model for an immune enhancer. Here, we focus on the modulation mechanism of 2-APB-induced *I*_*p*_ on STIM1-activated Orai1 channels to reveal how STIM1-mediated gating can be enhanced by 2-APB and to benefit future immune enhancer development. To minimize complex actions of 2-APB at high concentration, we adopted low dose of 2-APB (5 μM) throughout this study because this concentration can effectively potentiate STIM1-activated Orai1 current but not high enough to directly gate Orai3/Orai1 alone^18^.

The measured current density (I) is the multinomial product of the single-channel current (i), the number of active channels (N) and the open probability of the channels (*P*_*o*_). It was first postulated that 2-APB potentiated *I*_*CRAC*_ by increasing the *P*_*o*_^15^. However, by adopting Sigworth’s equation and performing the nonstationary noise analysis, later the same group suggested that 2-APB enhanced *I*_*CRAC*_ by increasing N at constant *P*_*o*_^10^. In fact, the 2-APB-induced potentiation effect only occurs on fully developed *I*_*CRAC*_ after completely emptying Ca^2+^ stores, in the case where full STIM1-Orai1 interaction has been established. Some groups observed that 2-APB increased fluorescence resonance energy transfer efficiency between Orai1-CFP and STIM1-YFP, suggesting a potentiation mechanism due to enhanced interaction of Orai1 and STIM1^22^,^23^,^24^. In addition, since the STIM1-free Orai1-E106D mutant became susceptible to 2-APB direct gating, it was suggested that 2-APB facilitated CRAC channels by altering the pore architecture^18^. Taken together, the exact mechanism underlying how 2-APB potentiates STIM1-activated *I*_*CRAC*_ remains elusive.

In the current study, first we directly probed the pore diameter of CRAC channels and found that 5 μM 2-APB enlarged the pore size of Orai1 from 3.8 Å to 4.6 Å. Then, we investigated the amplitude of 2-APB-induced *I*_p_ conducted by different sizes of ions, i.e., Ca^2+^, Na^+^, and Cs^+^, on WT and mutant CRAC channels with different pore diameters in the presence of STIM1. Our data suggest that the dilation effect of 2-APB, ion size and pore diameter jointly determine the generation and amplitude of *I*_*p*_ on CRAC channels. Additionally, we found that *I*_*p*_ could be induced in open Orai1 in the absence of STIM1.

## Results

### 2-APB modulates pore property of STIM1-activated Orai1 channels

**Figure 1:**
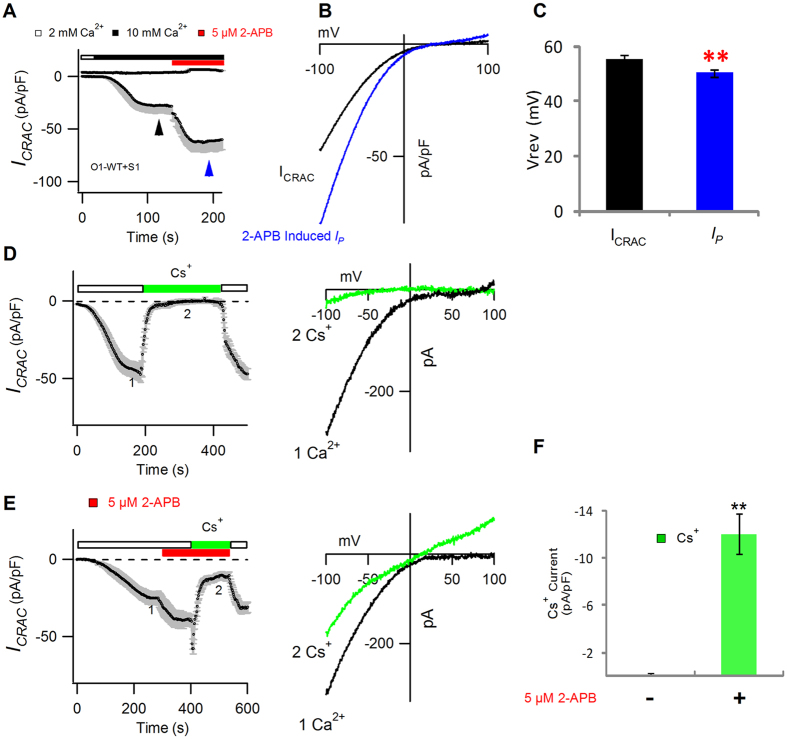
2-APB decreased *V*_*rev*_ and increased Cs^+^ permeability on STIM1-activated Orai1 channels. (**A**) Average *I*_*CRAC*_ evaluated at ± 100 mV in HEK293 cells co-expressing STIM1-mOrange2 and Orai1-mGFP. (**B**) *I-V* relationships of *I*_*CRAC*_ and *I*_*p*_. (**C**) The bar graph summarizes the *V*_*rev*_ of *I*_*CRAC*_ and *I*_*p*_. Values are mean ± SEM (n = 8). Compared with *I*_*CRAC*_, *I*_*p*_ displayed decreased *V*_*rev*_ (p = 0.008). (**D,E**) Average currents at −100 mV, plotted against time, as the extracellular solution was switched between 2 mM Ca^2+^ and Cs^+^-DVF solution on the WT CRAC channels (n = 7) (**D**) and 5 μM 2-APB-treated WT CRAC channels (n = 7) (**E**). *I–V* relationships of stable Ca^2+^*I*_*CRAC*_ (1, black) and stable Cs^+^ current (2, green) are shown to the right of panels (**D**,**E**), respectively. (**F**) Comparison of the amplitude of Cs^+^-conducted *I*_*CRAC*_ at −100 mV with or without 5 μM 2-APB. Values are mean ± SEM.

To reveal the underlying mechanism for 2-APB-induced *I*_*p*_, we analyzed the current amplitudes at −100 mV and +100 mV, *I-V* relationships, and *V*_*rev*_. Our data indicated that when a strong inward *I*_*p*_ was elicited by 5 μM 2-APB, a very small outward current (~1.8 ± 0.2 pA/pF) was observed at +100 mV (Fig. 1A,B). Consequentially, the *V*_*rev*_ of *I*_*CRAC*_ slightly shifted leftwardly from +55.5 mV to +50.4 mV after the application of 2-APB (Fig. 1C). This change in *V*_*rev*_ implied that 2-APB could alter the pore property slightly.

Cs^+^ permeability is frequently used to reflect the alteration of the CRAC channel pore property. On the WT CRAC channels, the Cs^+^-DVF solution could not conduct any detectable current (Fig. 1D). In contrast, 5 μM 2-APB pretreatment produced a small but sustained Cs^+^ current (−12.7 ± 1.4 pA/pF) (Fig. 1E), which was significantly bigger than that of the 2-APB-untreated CRAC channels (0.1 ± 0.1 pA/pF, p = 0.007) (Fig. 1F).

### 2-APB enlarges pore diameter of CRAC channels during potentiation

**Figure 2:**
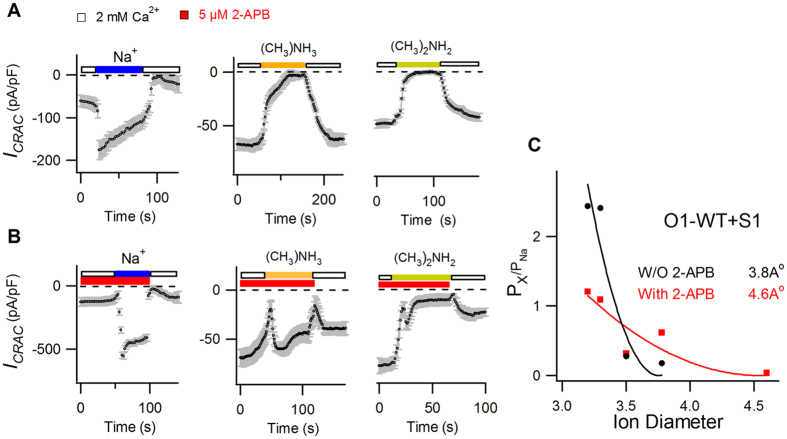
2-APB dilated pore diameter of CRAC channels during potentiation. (**A,B**) Average STIM1-activated WT Orai1 currents of Na^+^ and methylated ammonium monovalent cations, holding at −100 mV, are plotted against time in the absence (**A**) or presence (**B**) of 5 μM 2-APB (n = 6–10). (**C**) The P_x_/P_Na_ (X: ammonium and its methylated derivatives) is plotted against the pore diameter of each cation. The solid lines are fits to the hydrodynamic relationship. The estimated pore diameters of STIM1-activated Orai1 channels are 4.6 Å in the presence (red) and 3.8 Å in the absence (black) of 5 μM 2-APB.

The above results suggest that 2-APB could modulate the pore structure of CRAC channels. To directly probe the pore size of STIM1-activated Orai1 in the absence and presence of 2-APB, we estimated the narrowest region of channel pore by examining the permeation of a series of organic ammonium cations with different sizes^10^,^11^. In the absence of 2-APB, methylammonium (3.78 Å) and dimethylammonium (4.6 Å) could not conduct any detectable current on the WT CRAC channels (Fig. 2A). In contrast, 5 μM 2-APB considerably increased the permeability of these cations (Fig. 2B). The relative permeability of these cations over Na^+^ (P_x_/P_Na_) was calculated from the change of *V*_*rev*_ by using the Goldman-Hodgkin-Katz (GHK) voltage equation as reported previously^10^,^11^. By plotting the P_x_/P_Na_ against their respective ion diameters (Fig. 2C) and by fitting these values using the least square method following the hydrodynamic relationship^25^,^26^, we measured the narrowest region of the channel pore. Our estimated pore diameter of WT CRAC channels was 3.8 Å, identical to the previous reported value^10^,^11^, whereas the pore diameter of the 2-APB-treated CRAC channels was estimated to be 4.6 Å. Taken together, our data demonstrated that 2-APB altered the pore property and dilated the pore diameter of the WT CRAC channels.

### Pore-lining residue V102 of Orai1 affects 2-APB-induced *I*_*p*_

**Figure 3:**
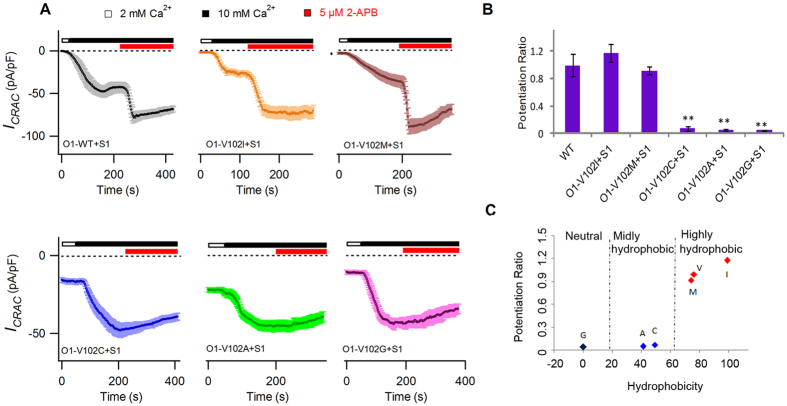
2-APB induced *I*_*p*_ on WT and V102X mutants. (**A**) Average *I*_*CRAC*_ evaluated at −100 mV. Currents of individual cells were corrected by subtracting the leak currents, normalized to cell size (pA/pF), averaged (n = 4–7) and plotted against time. Shortly after break-in, extracellular solution was shifted from 2 mM to 10 mM Ca^2+^. When *I*_*CRAC*_was fully developed and sustained, 5 μM 2-APB was applied. WT and V102I/M mutants displayed a prominent *I*_*p*_ current, while 2-APB failed to elicit *I*_*p*_ in V102 C/A/G mutants. (**B**) The bar graph summarizes the potentiation ratio (*I*_*p*_*/ I*_*CRAC*_) of WT and V102X mutants. Values are mean ± SEM. Compared with WT, V102C/A/G displayed significantly decreased potentiation ratios (V102C, p = 0.0013; V102A, p = 0.0002; V102G, p = 0.00005). (**C**) Dot plot of potentiation ratio against the hydrophobicity of side chains on residue 102. Red dots (V, I, M) represent channels with highly hydrophobic side chains on residue 102 and with high potency to generate *I*_*p*_. Blue dots (**A,C**) and black dot (**G**) denote channels with mildly hydrophobic or neutral side chains and without potency to generate *I*_*p*_.

V102 is a pore-lining residue of Orai1 that participates in the construction of the pore structure of CRAC channels^12^. It has been reported that V102C/A/G mutants yield constitutively active currents, even in the absence of STIM1, while WT and V102I/M mutants require STIM1 for activation^12^. Interestingly, we found that 5 μM 2-APB induced Ca^2+^ conducted *I*_*p*_ on STIM1-activated WT and V102I/M mutant channels, whereas *I*_*CRAC*_ of Orai1-V102C/A/G mutant channels could not be potentiated by the same dose of 2-APB (Fig. 3A,B).

2-APB-induced *I*_*p*_ has been shown to occur after store depletion^15^, the time when the CRAC channels have been fully activated by STIM1. One possibility for the disappearance of 2-APB-induced *I*_*p*_ on Orai1-V102C/A/G mutants could be the inadequacy of STIM1 activation. To test this possibility, we employed OSS constructs, which provides enough covalently linked functional STIM1 domains to fully activate the CRAC channels^12^,^27^. As shown in Supplementary Fig. S1, in contrast to OSS-WT/V102I/M, OSS-V102C/A/G could not yield 2-APB-induced *I*_*p*_. Therefore, even activated by enough STIM1 domains, Orai1-V102C/A/G mutants failed to generate 2-APB-induced *I*_*p*_.

Further analysis of the data revealed that the highly hydrophobic property of residues such as Val, Ile and Met correlated well with prominent 2-APB-induced *I*_*p*_; in contrast, substitution into neutral residue Gly or mildly hydrophobic residues such as Ala and Cys resulted in the disappearance of 2-APB-induced *I*_*p*_ (Fig. 3C). Therefore, the overall pattern indicated that the hydrophobic property of the side chain at residue 102 affected the generation of 2-APB-induced *I*_*p*_.

### Hydrophobicity of the side chain at residue 102 determines the pore diameter of CRAC channels

It has been reported that mutation of ion selectivity filter, such as E106D, alters the pore size and ion selectivity of the CRAC channels^11^. Because residue V102 is proposed to comprise a key hydrophobic gate in Orai1^12^, we assumed that STIM1-gated Orai1-V102C/A/G mutant channels might have altered pore properties compared with STIM1-gated WT and Orai1-V102I/M mutant channels, which might account for the disappearance of 2-APB-induced *I*_*p*_.

**Figure 4:**
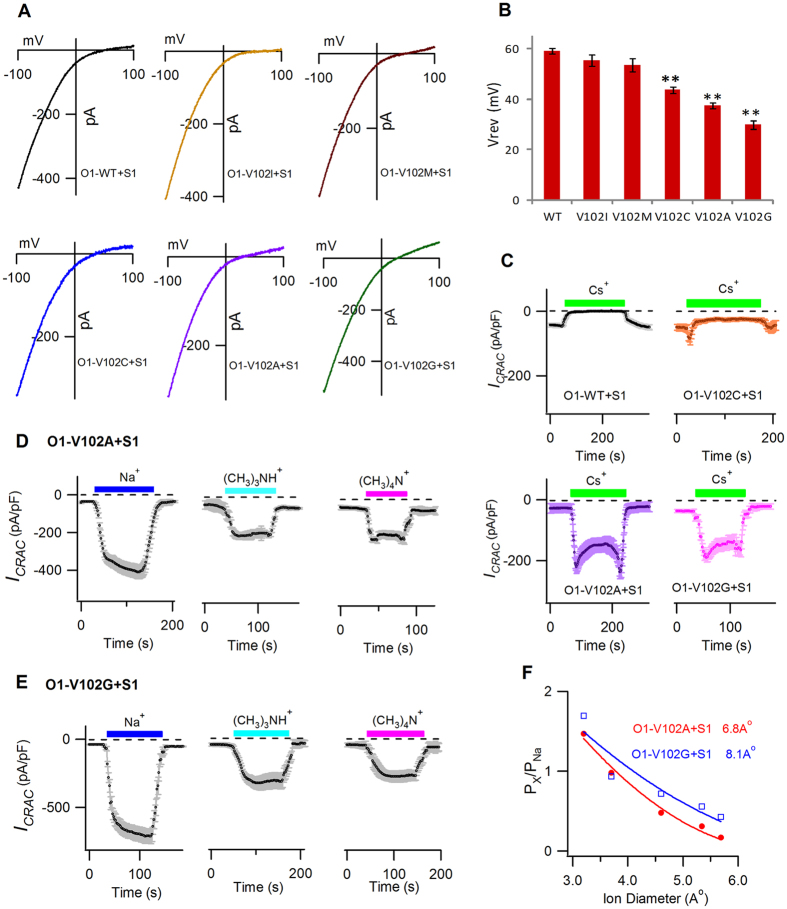
The *V*_*rev*_, Cs^+^ permeability and estimated pore diameters of V102X mutants. (**A**) *I–V* relationship (n = 6–10) of stable Ca^2+^ conducting *I*_*CRAC*_ in the WT channels and V102X mutants in the presence of STIM1. (**B**) The bar graph summarizes the *V*_*rev*_ values (mean ± SEM). Compared with WT, V102C/A/G displayed significantly decreased *V*_*rev*_ (V102C, p = 0.00018; V102A, p = 0.0000024; V102G, p = 0.0000001). No differences were observed between V102I/M mutants and the WT channels. (**C**) Mean currents evaluated at −100 mV, plotted against time (n = 6 each). The extracellular solution was switched between 2 mM Ca^2+^ and Cs^+^-based DVF solution. No Cs^+^ conduction was observed in the WT channels. In contrast, large Cs^+^ currents were recorded in V102A/G channels, while a small but significant Cs^+^ current was detected in V102C channels. (**D,E**) Mean currents of DVF-Na^+^ or DVF-methylated ammonium cations evaluated at −100 mV, plotted against time, on V102A channels (**D**) and V102G channels (**E**) in the presence of STIM1 (n = 6–10). The extracellular solution was switched between 2 mM Ca^2+^ and DVF solutions containing either Na^+^ or the indicated organic monovalent cations. Large methylated ammonium cations mediated significant currents on both V102A and V102G mutants. (**F**) P_x_/P_Na_ plotted against the pore diameter of each cation. The solid lines were fitted with least-squares method, following the hydrodynamic relationship. Estimated pore diameters were 6.8 Å and 8.1 Å for STIM1-activated V102A channels (red) and V102G channels (blue), respectively.

This assumption was first supported by the *V*_*rev*_ values of these two groups of STIM1-activated channels. As shown in Fig. 4A,B, Orai1-V102I/M mutants with highly hydrophobic side chains exhibited normal *V*_*rev*_ similar to the WT channels (WT, 59.3 ± 1.1 mV; V102M, 53.5 ± 2.7 mV; V102I, 55.6 ± 2.2 mV). In contrast, significantly decreased *V*_*rev*_ was observed in Orai1-V102X mutants with mildly hydrophobic and neutral amino acids (V102C, 43.8 ± 1.4 mV, p = 0.00018; V102A, 37.7 ± 1.2 mV, p = 0.0000024; V102G, 29.9 ± 1.6 mV, p = 0.0000001), which implied different pore properties between these two groups of channels.

Secondly, we examined Cs^+^ permeability between WT and V102C/A/G mutants activated by STIM1. In WT Orai1, *I*_*CRAC*_ was completely blocked by the Cs^+^-DVF solution. In contrast, the Cs^+^-DVF solution conducted prominent inward currents in Orai1-V102A/G mutants (V102A: −179.3 ± 9.8 pA/pF; V102G: −172.8 ± 13.5 pA/pF). The Orai1-V102C mutant had a much smaller Cs^+^ conducted inward current (−22.6 ± 4.8 pA/pF) than the V102A/G mutants, but it was still slightly larger than that of WT (Fig. 4C). Our data demonstrated that Orai1-V102C/A/G mutants with mildly hydrophobic or neutral side chains displayed increased Cs^+^ permeability compared with WT Orai1.

Finally, we estimated the pore diameter of STIM1-gated Orai1-V102A/G mutants. In contrast to the WT channels, these mutants could conduct larger monovalent cations, such as dimethylammonium, trimethylammonium (5.34 Å) and tetramethylammonium (5.6 Å) (Fig. 4D,E). The estimated pore diameters were 6.8 and 8.1 Å for STIM1-gated Orai1-V102A/G channels, respectively (Fig. 4F), larger than that of STIM1-gated Orai1-V102C channels (4.9 Å)^12^. To rule out the possibility that enlarged pore sizes could be caused by inadequacy of STIM1, we tested the permeability of tetramethylammonium ions (5.6 Å) on OSS-V102A/G mutants. As shown in Supplementary Fig. S2, our result indicated that tetramethylammonium ions could effectively pass through OSS-V102A/G mutants, confirming enlarged pore diameters (>5.6 Å) at saturated STIM1 binding for these Orai1 mutants.

Collectively, the leftward-shifted *V*_*rev*_, increased Cs^+^ permeability and larger pore diameters of STIM1-gated Orai1-V102C/A/G mutant channels proved that neutral or mildly hydrophobic side chains on 102 residues altered the channel pore properties and that these mutant CRAC channels were endowed with enlarged pore diameter. It should be noted that the gating of V102G channels may be different from that of V102C channels, because the introduction of a Gly may enhance flexibility of the pore helix at this site.

### Ion size and pore diameter jointly determine the amplitude of 2-APB induced *I*_*p*_

In light of the above results, we hypothesize that the steric hindrance between Ca^2+^ ions and the unusually narrow pore pathway of the WT CRAC channels slows the Ca^2+^ flowing speed and that the pore dilation effect of 2-APB decreases the steric hindrance, thus facilitates more Ca^2+^ passing through every single enlarged channel per unit time, which leads to the generation of *I*_*p*_. This hypothesis can explain why STIM1-gated Orai1-V102C/A/G could not yield 2-APB-induced *I*_*p*_. Because the pore diameters of these mutant channels (V102C: 4.9 Å, V102A: 6.8 Å, V102G: 8.1 Å) are much larger than that of the WT CRAC channels (3.8 Å), the steric hindrance between Ca^2+^ ions and pore pathway disappears; thus, Ca^2+^ can smoothly pass through these mutant channels without steric hindrance. Further dilation of the pore diameter by 2-APB cannot increase the Ca^2+^ flowing speed; therefore, no *I*_*p*_ can be observed in these mutant channels. According to this hypothesis, aside from the dilation action of 2-APB, the size of conducting ions and the pore diameter are two critical factors for the generation of *I*_*p*_.

To test this hypothesis, we selected two different sizes of monovalent cations, Na^+^ and Cs^+^, and adopted various STIM1-gated V102X mutant CRAC channels with different pore diameters; then, we checked the influence of ion size and pore diameter on the generation and amplitude of 2-APB induced *I*_*p*_.

**Figure 5:**
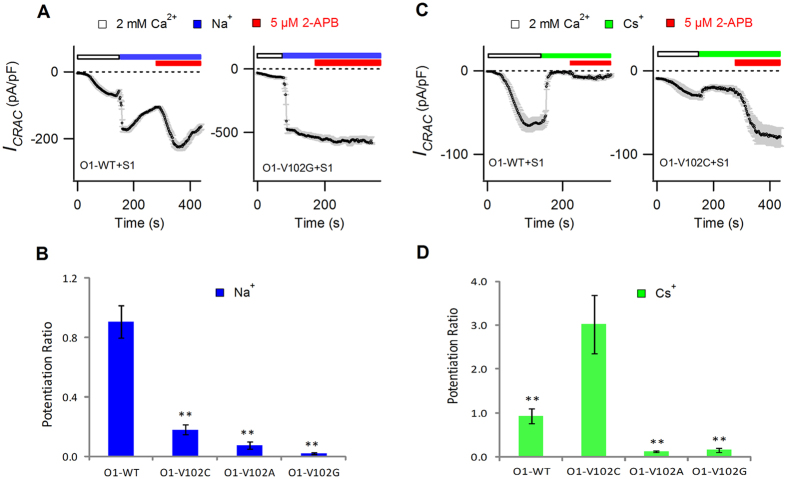
2-APB induced *I*_*p*_ was determined by both channel pore diameter and conducting ion size. Mean currents at −100 mV are plotted against time. Na^+^ or Cs^+^-based DVF solution was applied after achieving stable *I*_*CRAC*_ in 2 mM Ca^2+^ Ringer’s solution. (**A**) 5 μM 2-APB was applied on sustained Na^+^ current to elicit *I*_*p*_. (**B**) The bar graph summarizes the potentiation ratios of Na^+^ on WT and V102X mutants. Values are mean ± SEM (n = 4–6). The potentiation ratios of Na^+^ decreased significantly on all three mutants. **p < 0.01. (**C**) 5 μM 2-APB was applied on sustained Cs^+^ current to elicit *I*_*p*_. (**D**) The bar graph summarizes the potentiation ratios of Cs^+^ conducted *I*_*p*_ on WT and V102X mutants. Values are mean ± SEM (n = 4–6). Compared with WT with small pore diameter and V102A/G mutants with larger pore diameters, the V102C mutant had the highest potentiation ratio of Cs^+^.

The atomic diameter of a naked monovalent cation Na^+^ is 2.32 Å, very close to that of a naked divalent cation Ca^2+^, i.e., 2.28 Å^28^. Na^+^-DVF solution was applied after achieving stable *I*_*CRAC*_ in 2 mM Ca^2+^ Ringer’s solution. 2-APB was applied on sustained Na^+^ current to elicit *I*_*p*_. As shown in Fig. 5A,B, Na^+^ had a similar *I*_*p*_ pattern to Ca^2+^ and could conduct prominent 2-APB-induced *I*_*p*_ on the WT CRAC channels (−92.2 ± 10.2 pA/pF). However, the amplitude of Na^+^ *I*_*p*_ decreased significantly, along with the enlargement of pore diameter, i.e., on V102C/A mutants (V102C, −43.3 ± 11.5 pA/pF, p = 0.008; V102A, −34 ± 9.7 pA/pF, p = 0.004), and almost approached zero on the V102G mutant with the largest estimated pore diameter (−10.6 ± 3.2 pA/pF, p = 0.0001). To accurately reflect the relative extent of *I*_*p*_ over *I*_*CRAC*_, we calculated the potentiation ratio (*I*_*p*_/*I*_*CRAC*_), which decreased dramatically on all three mutants (V102C, 0.18 ± 0.03, p = 0.0002; V102A, 0.08 ± 0.02, p = 0.0003; V102G, 0.02 ± 0.007, p = 0.00004) compared with WT (0.91 ± 0.11).

The atomic diameter of a naked monovalent cation Cs^+^ is 3.8 Å^28^, which is larger than that of Ca^2+^. We tested Cs^+^ conducted *I*_*p*_ on STIM1-activated WT and V102C/A/G mutant channels. Our results indicated that Cs^+^ had a different *I*_*p*_ pattern compared with smaller ions such as Ca^2+^ and Na^+^ (Fig. 5C,D). The application of 2-APB on the WT channels elicited a very small Cs^+^*I*_*p*_ (−2.2 ± 0.2 pA/pF). As Orai1 pore became larger, Cs^+^-conducted *I*_*CRAC*_ gradually increased from −23.6 ± 5.0 pA/pF for V102C mutant to −182.2 ± 29.2 pA/pF for V102A mutant; however, 2-APB-induced *I*_*p*_ was decreased from −66.0 ± 12.0 pA/pF to −21.1 ± 3.7 pA/pF, correspondingly. And, further increases of the pore diameter in the V102G mutant failed to augment Cs^+^-conducted *I*_*p*_ (−15.9 ± 3.2 pA/pF). In contrast to Na^+^*I*_*p*_, the Cs^+^ potentiation ratio was highest for V102C mutant (3.02 ± 0.67) compared with that of WT (0.93 ± 0.17, p = 0.03) and the V102A/G mutant channels (V102A, 0.12 ± 0.02, p = 0.001; V102G, 0.16 ± 0.04, p = 0.001).

Taken together, ions with different sizes have different amplitudes of *I*_*p*_ and potentiation ratios on the same channel, and channels with different pore diameters generate different amplitudes of *I*_*p*_ and potentiation ratios when conducted by the same monovalent ion. Hence, whether *I*_*p*_ can be elicited by 2-APB and the amplitude of *I*_*p*_ are determined by both the channel pore diameter and the size of conducting ions.

### The generation of *I*_*p*_ by 2-APB is independent of STIM1

**Figure 6:**
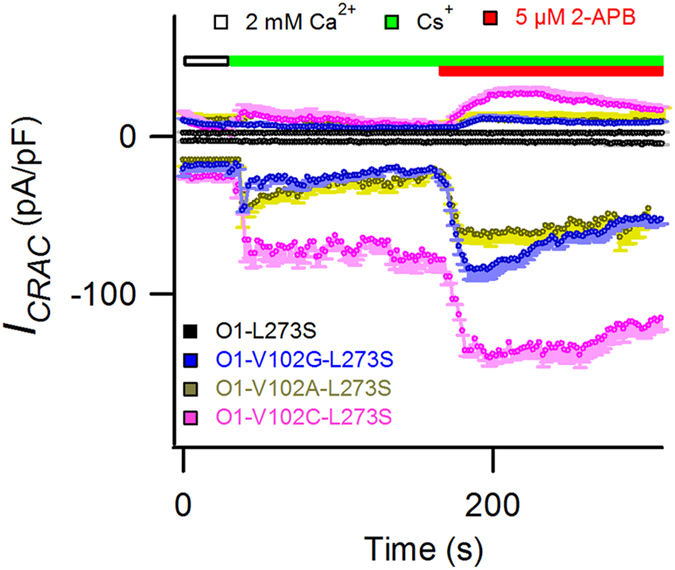
2-APB-induced Cs^+^ mediated *I*_*p*_ on Orai1-V102C/A/G-L273S mutants. Time course of average currents recorded at ± 100 mV in HEK293 cells transiently expressing WT Orai1-L273S or V102C/A/G-L273S mutants in the absence of STIM1. The inwardly rectified currents were corrected for leak currents, normalized with the cell sizes, averaged (n = 5 each) and plotted against time. Cs^+^-based DVF solution was applied after achieving stable, constitutively active currents in 2 mM Ca^2+^ Ringer’s solution; 5 μM 2-APB was applied on sustained Cs^+^ current to elicit *I*_*p*_. On the WT channels, 2-APB failed to elicit any detectable Cs^+^ current, while prominent Cs^+^*I*_*p*_ were observed on the 2-APB-treated Orai1-V102C/A/G-L273S mutants.

Our above results were obtained in the presence of STIM1. Because a high dose of 2-APB could directly gate Orai3, marginally gate STIM1-free Orai1, and generate inwardly and outwardly rectified current in a STIM1-independent manner^16^,^18^, we further tested the involvement of STIM1 in 2-APB induced *I*_*p*_ on Orai1. We expressed Orai1-V102C/A/G-L273S mutants in the absence of STIM1. V102C/A/G mutation was employed to keep Orai1 in an open state. And L273S mutation eliminates the interaction between expressed Orai1 and endogenous STIM1 in HEK293 cells^29^. Our data indicated that without the involvement of STIM1, the WT Orai1 channels conducted little Cs^+^ current in the presence of 2-APB; together with a previous study^18^, these results suggest that closed Orai1 alone is largely unresponsive to 2-APB. In contrast, 2-APB could further potentiate Cs^+^ current on constitutively active Orai1-V102C/A/G-L273S channels (Fig. 6). Furthermore, the EC_50_ for 2-APB enhanced current on the Orai1-V102A-L273S mutant is 5.4 μM (Supplementary Fig S3); this value is close to the reported EC_50_ for 2-APB-induced *I*_*p*_ on STIM1-activated Orai1 (4 μM) and is much smaller than the reported EC_50_ for direct gating of 2-APB on Orai1 alone (20 ± 1 μM)^18^. Hence, our results suggest that 2-APB at 5 μM concentration potentiated rather than directly gated Orai1-V102A-L273S channels, and the generation of *I*_*p*_ requires the open state of the CRAC channels which are either activated by STIM1-binding or constitutively generated by V102A/C/G mutants in the absence of STIM1.

## Discussion

**Figure 7:**
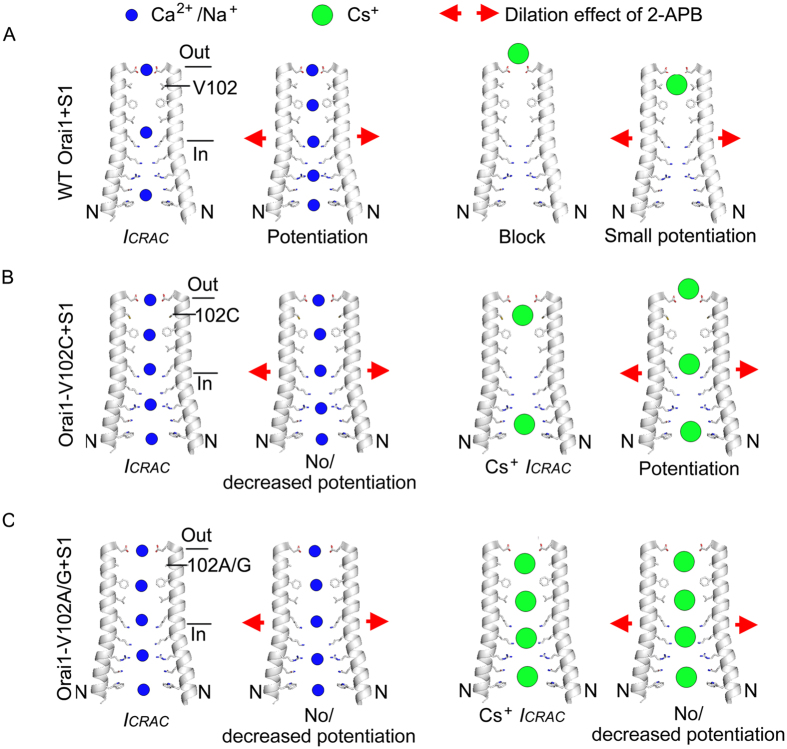
The schematic model for the generation of 2-APB-induced *I*_*p*_. Two gray M1 helices are employed to indicate the channel pore structure as described previously^30^. Red arrows represent the pore dilation effect of 2-APB. Blue and green dots denote small Ca^2+^/Na^+^ ions and large Cs^+^ ions, respectively. More dots in channel pore represent increased flowing speed. (**A**) On STIM1-activated WT Orai1 channel with small pore diameter of 3.8 Å, once 2-APB dilates the pore diameter to 4.6 Å, more Ca^2+^/Na^+^ ions flowing through the channel pore generates *I*_*p*_. Large Cs^+^ ions block the pore, 2-APB-induced dilation facilitates the passing of Cs^+^, and a small Cs^+^*I*_*p*_ is observed. (**B**) On STIM1-activated Orai1-V102C mutant channel with an intermediate pore diameter (4.9 Å), Ca^2+^/Na^+^ can pass through the channel at highest speed in the absence of 2-APB; hence, little *I*_*p*_ emerges after 2-APB application. Whereas large Cs^+^ ions cannot pass through the channel at maximum speed, further dilation of the pore by 2-APB induces prominent Cs^+^*I*_*p*_. (**C**) On STIM1-activated Orai1-V102A/G mutant channels with a large pore diameter (6.8 Å and 8.1 Å, respectively), both Ca^2+^/Na^+^ and Cs^+^ ions could pass through the channel at maximum speed. Hence, no *I*_*p*_ could be elicited by 2-APB.

In this study, we have investigated the underlying mechanism for 2-APB-elicited *I*_*p*_ by directly probing the pore diameter of CRAC channels and understanding how ion size and channel pore diameter contribute to the generation and amplitude of *I*_*p*_. Our data demonstrate that 2-APB directly dilates the pore of open Orai1 in the presence of STIM1, increasing the pore diameter from 3.8 Å to 4.6 Å. Ions with small sizes, such as Ca^2+^ and Na^+^, mediate prominent 2-APB-induced *I*_*p*_ on the WT CRAC channels and conduct no or decreased *I*_*p*_ on V102C/A/G mutant channels with enlarged pore diameters. On the contrary, large Cs^+^ ions, blocking the WT CRAC channels, is capable of conducting prominent *I*_*p*_ on the pore-enlarged V102C mutant. Therefore, we conclude that the main cause of *I*_*p*_ is the pore dilation effect of 2-APB. Additionally, the size of conducting ions and the channel pore diameter both contribute to the generation and amplitude of 2-APB-elicited *I*_*p*_. We summarize these results in a schematic model (Fig. 7) to explain how these three factors jointly determine *I*_*p*_.

The unusually narrow CRAC channel pore pathway comprises a glutamate ring, hydrophobic section and basic region^30^. Therefore, cations will encounter steric hindrance and a large energy barrier provided by Vander Waals interaction and electrostatic repulsion along the permeation pathway. The generation and amplitude of *I*_*p*_ depend on two factors: 1) whether hindrance exists between cations and pore pathway, and 2) whether hindrance could be overcome by the pore-dilation effect of 2-APB. No Na^+^- or Ca^2+^-conducting *I*_*p*_ is observed on the pore-enlarged V102C/A/G mutant channels, probably due to the lack of hindrance between small Na^+^/Ca^2+^ and enlarged pore. Very small Cs^+^-conducting *I*_*p*_ is detected in the WT CRAC channels, probably because the hindrance between large Cs^+^ and small channel pore could not be effectively overcome by the dilation effect of 2-APB. However, when the pore size becomes larger on Orai1-V102C mutant, pore-dilation effect of 2-APB induces prominent Cs^+^*I*_*p*_. Our data strongly suggest that 2-APB facilitates more ions passing through every single channel per unit time via the pore dilation effect, generates larger *I*_*p*_ by increasing i rather than *P*_*o*_or N. To directly validate this hypothesis, we performed nonstationary noise analysis of 2-APB effect on native (Jurkat cells) as well as recombinant (Orai1 and STIM1 transfected HEK293 cells) CRAC channels employed the same protocol as previously reported^20^,^31^. As demonstrated in Supplementary Fig. S4, on Jurkat cells, the mean unitary Na^+^ current was significantly raised from to 90.5 ± 2.0 fA to 241.5 ± 9.1 fA (n = 5 cells for each group, p = 0.0000002). On STIM1-gated Orai1 channels in HEK293 cells, 2-APB application increased the mean unitary Na^+^ current from 85.0 ± 2.2 fA (n = 3 cells) to 245.8 ± 4.3 fA (n = 4 cells, p = 0.000001). No significant alteration of *P*_*o*_ or N was observed.

2-APB exerts complex effects on CRAC channels: potentiation of STIM1-activated *I*_*CRAC*_ at low concentration, inhibition of it at high concentration, and direct gating of Orai1 and Orai3 in the absence of STIM1 at high concentration. The fact that potentiation, inhibition and direct gating have different EC_50_ suggests multiple action sites of 2-APB on Orai1 and/or STIM1 proteins^18^. Previous studies have shed some lights on the molecular mechanisms of direct gating effect of 2-APB. It’s reported that 2-APB directly gated Orai3 and enlarged its pore diameter^16^,^18^,^19^,^20^. On Orai3, the action site of 2-APB has been attributed to the TM2-TM3 region, somewhere near C101/G158 residues^32^,^33^. It is tempting to speculate that 2-APB at low dose could bind to a similar site in TM2-TM3 region in close Orai1 channel but it is not sufficient to directly open the channel, whereas conformational changes of open Orai1 may: 1) lower the energy barrier for 2-APB to further dilate the channel; or 2) create a new, high affinity binding site for 2-APB to further dilate the channel pore, which then results in *I*_*p*_. As more 2-APB molecules interact with CRAC channels, probably at other site(s), the channels may transit into a closed state.

In contrast to the agonistic activation of closed Orai3, low concentration of 2-APB could act as an enhancer on open Orai1 at the right time, thus avoiding aberrant activation of immune cells in a resting state. 2-APB induced *I*_*p*_ on STIM1-gated Orai1 has an inwardly rectified *I-V* profile, with little outward current at + 100 mV (Fig. 1B), quite distinct from 2-APB-activated Orai3 current^16^,^18^. The resulting 2-APB induced *I*_*p*_ on Orai1 largely resembles the properties of native *I*_*CRAC*_, preventing aberrant ion signals from interfering with the normal immune reaction. Hence, both STIM1-mediated gating can be enhanced and high Ca^2+^ selectivity of *I*_*crac*_ can be sustained by the pore-dilation effect of 2-APB; as an ideal model for an immune enhancer, the activity-dependent potentiation effect of 2-APB on Orai1 should be employed in the development of specific immune enhancers.

Modulation of ion channels is a powerful approach to altering physiological responses for therapeutic benefit. In the future, it will be theoretically feasible to develop a potential immune enhancer like 2-APB that specifically potentiates native CRAC channels, but without its inhibitory effect. The challenge remains to delineate the action sites of 2-APB and how 2-APB binding alters the gating and pore structure of Orai1. Further work involving structural studies and the site-directed mutagenesis of CRAC channel proteins should help to solve this challenge.

## Methods

### Cell Culture and Transfection

HEK293 cells (ATCC) were cultured in Dulbecco’s modified Eagle’s medium (DMEM) with 10% heat-inactivated fetal bovine serum (FBS), 50 U/ml penicillin and 50 mg/ml streptomycin. Cells in one well of 6-well plate were transfected with 2 μg DNA using Lipofectamine 2000 reagent (Invitrogen). If the constructs could generate constitutive active *I*_*CRAC*_, for example, Orai1-V102C/A/G mutants, customized Ca^2+^ free DMEM medium (Merck) with 10% FBS was used to culture cells to avoid the toxicity induced by spontaneous Ca^2+^ influx. For coexpression experiments, Stim1-mOrange2 and Orai1-mGFP (WT or mutant) were co-transfected at a ratio of 2:1. The positive transfected cells were selected by green and red fluorescence.

### Plasmids Construction

The STIM1-mOrange2 and Orai1-mGFP plasmids have been described previously^34^,^35^. Site-directed mutagenesis was performed by PCR-driven overlap extension and confirmed by sequencing. For the construction of Orai1-V102I/M/C/A/G-mGFP, pmGFP-N1-Orai1 (Orai1-mGFP) served as a template. We designed primers with the Primer Premier 5.0 software, amplified the upstream and downstream fragments of human *ORAI1* by using mutagenic primers and flanking primers, fused two fragments together by overlap PCR, digested with XhoI (5′) and BamH1 (3′) and inserted them into a pmGFP-N1 vector. To construct Orai1-V102C/A/G-L273S-mGFP, we additionally mutated L273S on the basis of Orai1-V102X-mGFP.

### Solutions and Chemicals

All solutions were prepared as described previously^11^. The standard extracellular Ringer’s solution contained (in mM) 120 NaCl, 2 MgCl_2_ 10 TEACL, 10 HEPES, 10 CaCl_2_, and 10 D-glucose (pH adjusted to 7.4 using NaOH). The standard divalent free solution (DVF) contained (in mM) 152 NaCl, 10 TEACL, 10 HEDTA, 10 HEPES, and 1 EDTA (pH adjusted to 7.4 using N-methyl-D-glucamine (NMDG) hydroxide). Where indicated, the following organic ammonium derivatives were substituted for NaCl in the standard DVF solution: ammonium chloride (NH_4_Cl), methylamine HCl (CH_3_ NH_2_-HCl), dimethylamine HCl ((CH_3_)_2_NH-HCl), trimethyl-amine HCl ((CH_3_)3N-HCl), tetramethylammonium chloride ((CH_3_)_4_NCl), hydroxylamine HCl (NH_2_OH-HCl), and hydrazine HCl (NH_2_NH_2_-HCl). NMDG was used to adjust pH to 7.4, except in the cases of hydrazine HCl (pH 6.4) and hydroxylamine HCl (pH 6.2), which were studied at acidic pH to increase the ionized concentration of the test ion. The standard intracellular solution contained (in mM) 120 Cesium-glutamate, 8 MgCl_2_, 10 BAPTA, 10 HEPES (pH adjusted to 7.2 using CsOH). Unless noted otherwise, all chemicals, including 2-APB, were purchased from Sigma-Aldrich.

### Electrophysiology and Data analysis

Patch clamp experiments were performed at 21–25 °C using the standard whole-cell recording configuration as previously described^34^. Only cells with high input resistance (>2 Ω) were selected for recording; membrane potentials were corrected for a liquid junction potential of 10 mV. The holding potential was set to 0 mV, and currents were monitored by voltage ramps of 50 ms, spanning a range of −100 to + 100 mV, applied at 2 s intervals over a period of 100–400 s. Currents were filtered at 2.9 kHz and digitized at a rate of 20 kHz. Currents obtained before the activation of CRAC channels were assigned as leak currents and subtracted from the subsequent recorded currents. On constitutively active channels, the accurate current or *V*_*rev*_ value was measured by correcting the leak currents collected in Ca^2 + ^ Ringer’s solution with 50 μM LaCl_3_. Average currents are presented as mean ± SEM. All patch clamp data analysis and curve fitting was done with Igor Pro 5.03 (WaveMetrics). The potentiation ratios were calculated by dividing the *I*_*p*_ by corresponding *I*_*CRAC*_ (*I*_*p*_*/I*_*CRAC*_) of an individual cell. The relative permeability of different cations was calculated from the change in reverse potential by using the GHK equation as previously described^10^,^11^. The p value was calculated by two-tailed t-test.

## Additional Information

**How to cite this article**: Xu, X. *et al*. 2-Aminoethoxydiphenyl Borate Potentiates CRAC Current by Directly Dilating the Pore of Open Orai1. *Sci. Rep.* **6**, 29304; doi: 10.1038/srep29304 (2016).

## Supplementary Material

Supplementary Information
