# Supplementary material for: 2-Aminoethoxydiphenyl Borate Potentiates CRAC Current by Directly Dilating the Pore of Open Orai1
Source: Sci Rep. 2016 Jul 4;6:29304. doi: 10.1038/srep29304 (PMC4931693; doi:10.1038/srep29304)
Supplement: Supplementary Information [file srep29304-s1.pdf]

2-Aminoethoxydiphenyl Borate Potentiates CRAC Current by Directly Dilating the Pore of Open Orai1

Xiaolan Xu[1,+], Sher Ali[1,2,+], Yufeng Li[1,3], Haijie Yu[4], Mingshu Zhang[5], Jingze Lu[1], Tao XU[1]

[1] National Key Laboratory of Biomacromolecules, Institute of Biophysics, Chinese Academy of Sciences, Beijing 100101, China.

[2] University of Chinese Academy of Science, Beijing 100049, China.

[3] College of Life Science, Sichuan Normal University, Chengdu 610101, China.

[4] Department of Physiology and Biophysics, University of Washington, Seattle, WA, USA.

[5] Key Laboratory of RNA Biology, Institute of Biophysics, Chinese Academy of Sciences, Beijing 100101, China.

[+] These authors contributed equally to this work.

Correspondence and requests for materials should be addressed to T.X. (email: xutao@ibp.ac.cn)

Supplementary Methods

Plasmids construction

The OSS plasmid has been described previously[1]. For the construction of OSS-V102I/M/C/A/G-mGFP, we eliminated the recognition site for restriction enzyme HindIII from the *ORAI1* gene by synonymous mutation, then adopted HindIII-free *ORAI1* as a template, performed overlap PCR, digested with HindIII (5') and Kpn1 (3'), inserted into the same restriction enzyme digested pmGFP-N1-OSS vector. All mutants were confirmed by sequencing.

Jurkat cells

Jurkat E6.1 T cells (ATCC) were grown in RPMI 1640 medium supplemented with 10% FBS, 2 mM glutamine.

Noise analysis

The nonstationary noise analysis was performed as introduced previously[2-4]. For noise analysis experiments, $CaCl_2$ was added to the standard Na^+ DVF solution at an appropriate amount calculated from Max-Chelator software (WEBMAXC 2.10, available at http://www.stanford.edu/~cpatton/webmaxc2.htm). 200-ms sweeps were acquired at the rate of 4 Hz at the holding potential of -100 mV, digitalized at 20 KHz, currents were low pass-filtered at 1 kHz. The mean current and variance were calculated from a 200-ms segment. The single-channel current (i) was calculated as reported previously[2-4].

Supplementary Figures:

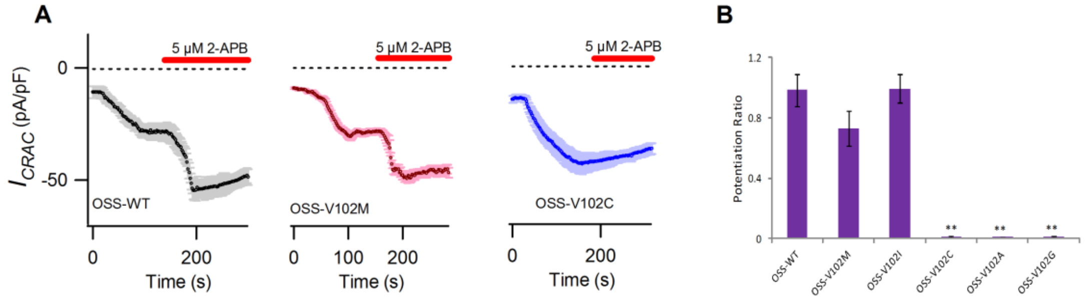

Supplementary Fig. S1. 2-APB induced Ca^{2+} conducted I_p on OSS-WT and OSS-V102X mutants. (A) Time course of the development of average I_{CRAC} evaluated at -100 mV in HEK293 cells transiently expressing OSS-WT and representative OSS-V102M/C mutants. Constitutively active currents were detected at break-in. Currents of each individual cell were corrected for leak currents, normalized to cell sizes, averaged (n=5 each) and plotted against time. Extracellular Ringer's solution was shifted from 2 mM Ca^{2+} to 10 mM Ca^{2+} soon after break-in. When I_{CRAC} was fully developed and sustained, 5 μM 2-APB was applied and induced prominent I_p on OSS-WT and OSS-V102M, while it failed to elicit I_p on the OSS-V102C mutant. (B) The bar graph summarizes the potentiation ratio (I_p/ I_{CRAC}) of OSS-WT and OSS-V102X mutants. Values are mean ± SEM (n=5 each). Compared with WT, OSS-V102C/A/G displayed significantly decreased potentiation ratio (OSS-V102C, p=0.00002; OSS-V102A, p=0.00002; OSS-V102G, p=0.00003). No significant differences were observed between OSS-V102M/I mutants and OSS-WT channels.

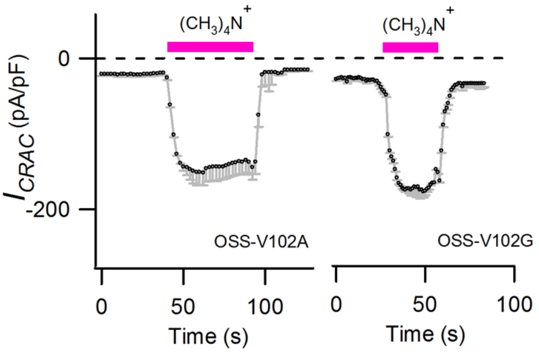

Supplementary Fig. S2. The permeability of tetramethylammonium ions on OSS-V102A/G mutants. Mean currents evaluated at −100 mV, plotted against time (n=5 each). The extracellular solution was switched between 2 mM Ca2+ and tetramethylammonium cations-based DVF solution. Large tetramethylammonium ions mediated significant currents on OSS-V102A/G mutants.

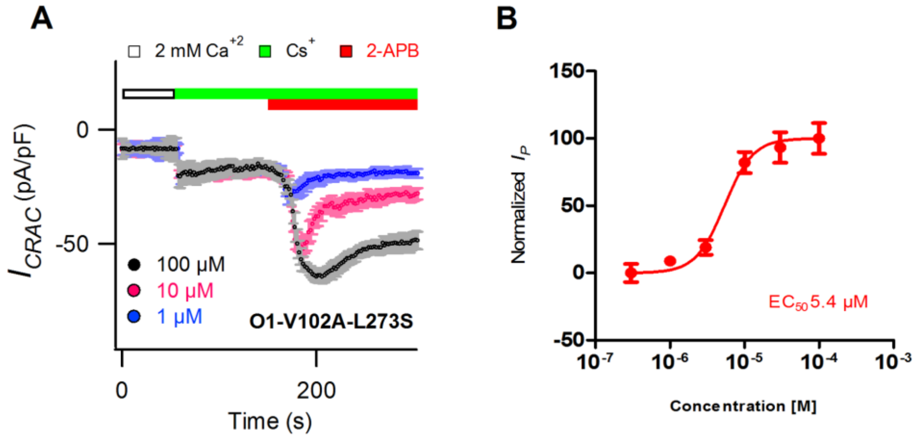

Supplementary Fig. S3. EC_{50} for 2-APB enhanced currents on Orai1-V102A-L273S mutant. (A) Time course of the development of average current recorded at -100 mV in HEK293 cells transiently expressing the V102A-L273S mutant in the absence of STIM1. The inwardly rectified currents were corrected for leak currents, normalized with the cell sizes, averaged (n=6 each) and plotted against time. Cs^+-based DVF solution was employed after achieving stable constitutively active currents in 2 mM Ca^{2+} Ringer's solution. Various doses of 2-APB were applied on sustained Cs^+ current to elicit I_p. (B) Dose response of 2-APB on Orai1-V102A-L273S mutant. The EC_{50} for the potentiation was 5.4 µM.

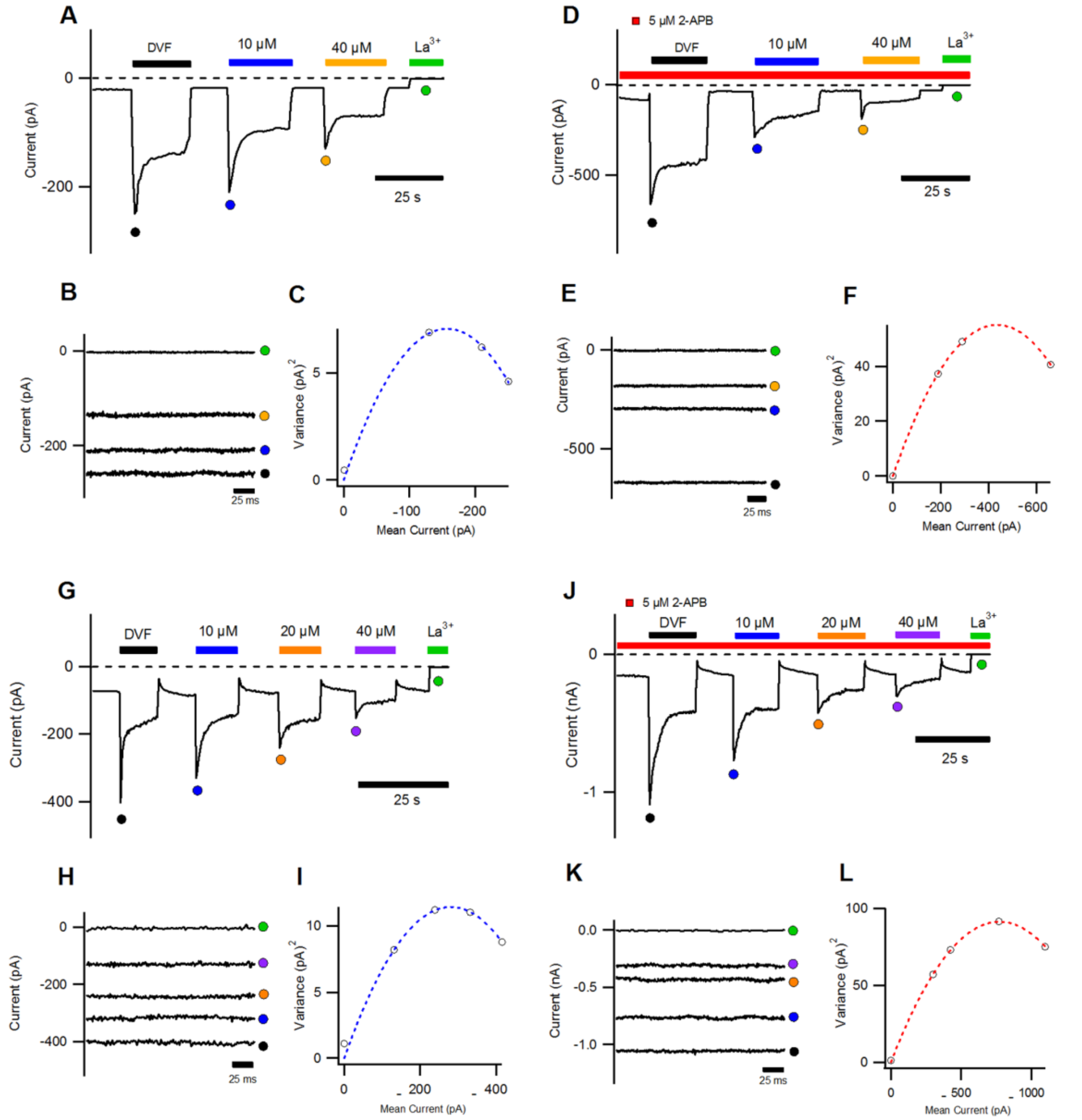

Supplementary Fig. S4. Estimates of the unitary Na^+ current on native and recombinant CRAC channels in the absence and presence of 5 μM 2-APB. (A-C) Noise analysis of CRAC currents on a Jurkat cell in the absence of 2-APB. (A) A TG-pretreated Jurkat cell was held at a constant potential of -100 mV. The bars indicate the periodic exchange of DVF solutions containing various concentration of $[Ca^{2+}]_o$ with 2 mM $[Ca^{2+}]_o$ Ringer solution. The background current and variance were collected in 2 mM Ca^{2+} with 50 μM La^{3+}. (B) For each application of Na^+ DVF solution without or with various $[Ca^{2+}]_0$, a single trace of 200-ms was collected as indicated by colored dots at the peak of Na^+ current, and was divided into 25-ms segments for the analysis. (C) Variance versus current plots were fitted by parabolic function to calculate the unitary Na^+ current. The blue dashed line is a fit with i = 89.9 fA, N = 3,493 channels, and with these values, the estimated P_o in Na^+-DVF is 0.79. (D-F) On a Jurkat cell in the presence of 5 μM 2-APB. The red bar indicates the application of 5 μM 2-APB. The fitting result of the red dashed line is i = 258.9 fA, N = 3,453 channels, and P_o = 0.75 in Na^+-DVF. (G-I) On Orai1 and STIM1 transfected HEK293 cell in the absence of 2-APB. The blue dashed line is a fit with i = 81.5 fA, N = 6,889 channels, and P_o = 0.72 in Na^+-DVF. (J-L) On Orai1 and STIM1 transfected HEK293 cell in the presence of 5 μM 2-APB.

The fitting result of the red dashed line is i = 236.3 fA, N = 6,542 channels, and P_o = 0.70 in Na^+-DVF. On Jurkat cells, the mean unitary Na^+ current was significantly raised from to 90.5 ± 2.0 fA to 241.5 ± 9.1 fA (n=5 cells for each group, p=0.0000002). On STIM1-gated Orai1 channels in HEK293 cells, 2-APB application increased the mean unitary Na^+ current from 85.0 ± 2.2 fA (n=3 cells) to 245.8 ± 4.3 fA (n=4 cells, p=0.000001). 2-APB application did not cause significant alteration on P_o or N either on native (P_o, p=0.24; N, p=0.11) or on recombinant CRAC channels (P_o, p=0.13; N, p=0.88).

References:

1 Li, Z. *et al.* Graded activation of CRAC channel by binding of different numbers of STIM1 to Orai1 subunits. *Cell Res* **21**, 305-315 (2011).

2 Mullins, F. M. & Lewis, R. S. The inactivation domain of STIM1 is functionally coupled with the Orai1 pore to enable Ca^{2+}-dependent inactivation. *J Gen Physiol* **147**, 153-164 (2016).

3 Prakriya, M. & Lewis, R. S. Regulation of CRAC channel activity by recruitment of silent channels to a high open-probability gating mode. *J Gen Physiol* **128**, 373-386 (2006).

4 Yamashita, M. & Prakriya, M. Divergence of Ca^{2+} selectivity and equilibrium Ca^{2+} blockade in a Ca^{2+} release-activated Ca^{2+} channel. *J Gen Physiol* **143**, 325-343 (2014).
